# Bi-allelic mutations in *uncoordinated mutant number-45 myosin chaperone B* are a cause for congenital myopathy

**DOI:** 10.1186/s40478-019-0869-1

**Published:** 2019-12-18

**Authors:** Hormos Salimi Dafsari, Nur Mehpare Kocaturk, Hülya-Sevcan Daimagüler, Anna Brunn, Jörg Dötsch, Joachim Weis, Martina Deckert, Sebahattin Cirak

**Affiliations:** 1 Department of Pediatrics, Faculty of Medicine and University Hospital Cologne, University of Cologne, 50937 Cologne, Germany; 2 Center for Molecular Medicine (CMMC), Faculty of Medicine and University Hospital Cologne, University of Cologne, 50937 Cologne, Germany; 3 Institute of Neuropathology, Faculty of Medicine and University Hospital Cologne, University of Cologne, 50931 Cologne, Germany; 4 Center for Rare Diseases, Faculty of Medicine and University Hospital Cologne, University of Cologne, 50937 Cologne, Germany; 5 Institute of Neuropathology, RWTH University Hospital, 52074 Aachen, Germany

## Abstract

Congenital myopathies (CM) form a genetically heterogeneous group of disorders characterized by perinatal muscle weakness. Here, we report an 11-year old male offspring of consanguineous parents of Lebanese origin. He presented with proximal weakness including Gower’s sign, and skeletal muscle biopsy revealed myopathic changes with core-like structures. Whole exome sequencing of this index patient lead to the discovery of a novel genetically defined CM subtype based on bi-allelic mutations in the uncoordinated mutant number-45 myosin chaperone B (UNC45B) NM_173167:c.2261G > A, p.Arg754Gln. The mutation is conserved in evolution and co-segregates within the pedigree with the phenotype, and located in the myosin binding armadillo repeat domain 3 (ARM3), and has a CADD Score of 35. On a multimeric level, UNC45B aggregates to a chain which serves as an assembly line and functions as a “template” defining the geometry, regularity, and periodicity of myosin arranged into muscle thick filaments. Our discovery is in line with the previously described myopathological phenotypes in *C. elegans* and in vertebrate mutants and knockdown–models. In conclusion, we here report for the first time a patient with an UNC45B mutation causing a novel genetically defined congenital myopathy disease entity.

## Text

**Fig. 1:**
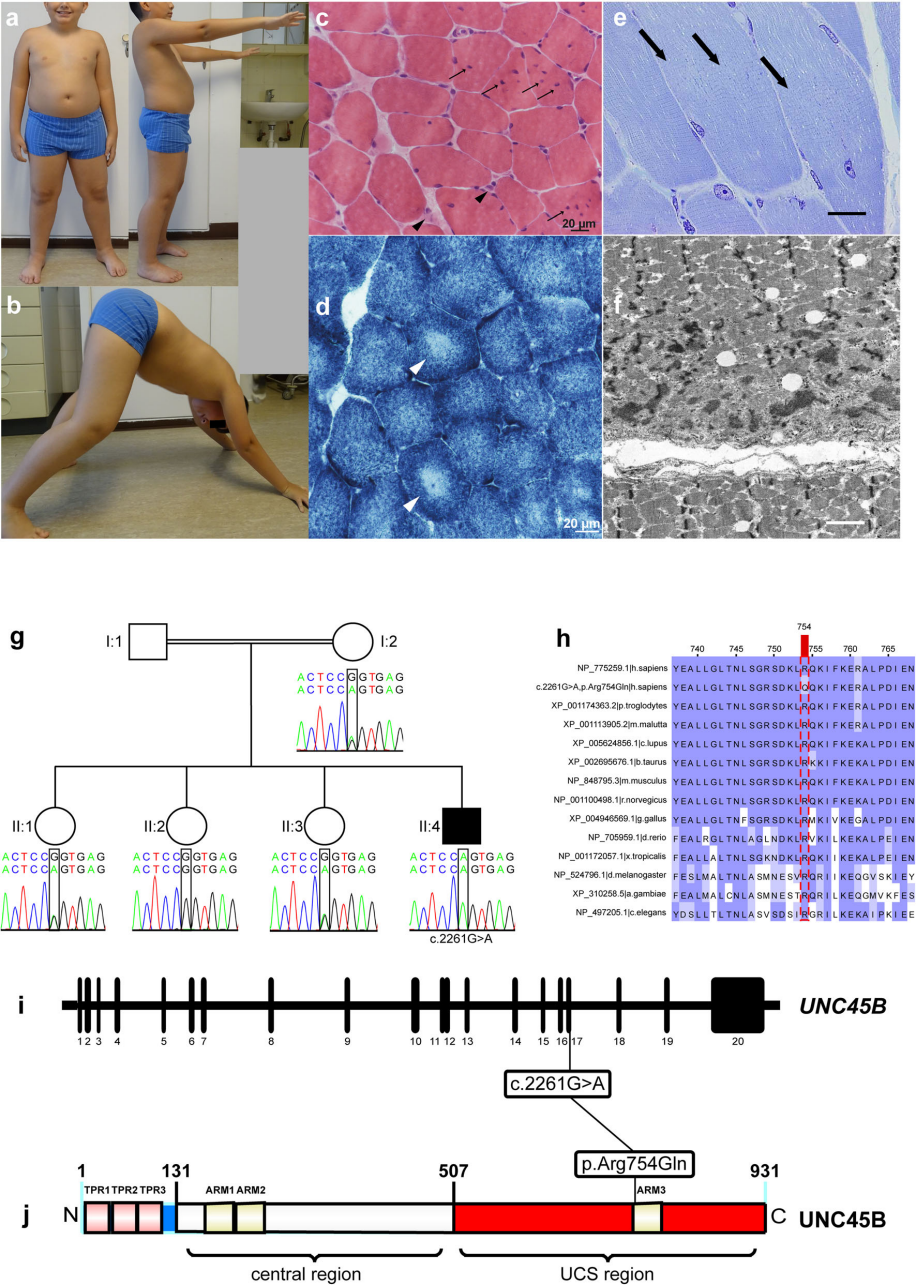
Phenotypic, myopathological, and electron microscopical findings in our patient with an overview of the *UNC45B* variant identified in this study. **a** Front and side view of our patient, showing hyperlordosis and obesity. **b** Gower’s sign in our patient. **c** Increased fiber size caliber spectrum with internalized nuclei predominantly in hypertrophic muscle fibers (arrows) and atrophic muscle fibers (arrowheads). H&E staining, original magnification × 400; scale bar 20 μm. **d** Disorganization of myofibrillary architecture evidenced by pale centers of muscle fibers presenting as core-like structures. Enzyme histochemistry with NADH; original magnification × 400; scale bar 20 μm. **e** Focal myofibrillary disintegration (arrows) and occasional non-subsarcolemmal muscle fiber nuclei. Semithin section, toluidine blue staining; scale bar 20 μm. **f** Subsarcolemmal core-like structure and Z-band streaming in electron microscopy (EM). **g** Pedigree and chromatograms of the index patient, healthy sisters, and healthy mother. The healthy father’s blood sample was unavailable for dideoxy sequencing. **h** UNC45B multiple sequence alignment made with Jalview shows high evolutionary conservation at amino acid residue p.Arg754 (NP_775259.1 *Homo sapiens*, the mutated sequence from our patient c.2261G > A p.Arg754Gln, XP_001174363.2 *P. troglodytes*, XP_0011113905.2 m. mulatta, XP_005624856.1 *C. lupus*, XP_002695676.1 *B. taurus*, NP_848795.3 m. musculus, NP_001100498.1 *R. norvegicus*, XP_004946569.1 g. gallus, NP_705959.1 *D. rerio*, NP_001172057.1 x. tropicalis, NP_524796.1 *D. melanogaster*, XP_310258.5 *A. gambiae*, and NP_497205.1 *C. elegans*). **i** Variant in the *UNC45B* gene (NM_173167.3, 20 exons) identified in our patients and concomitant position in the **j.** UNC45B protein structure (Q8IWX7) based on 931-aa isoform (ENST00000268876.9, NP_775259.1); pictogram with protein domains: Tetratricopeptide repeats (TPR, red) and Armadillo/beta-catenin-like repeats (ARM, green), N-terminal region of protein in blue, central region (131–506) in white, UCS region (Unc45−/Cro1p−/She4p-related protein) in red (507–931). Gene and protein sequences are drawn with the IBS Biocuckoo web server [14]

Congenital myopathies (CM) form a genetically heterogeneous group of disorders characterized by perinatal muscle weakness [18, 20]. Here, we report an 11-year old male offspring of consanguineous parents of Lebanese origin. He presented with proximal weakness including Gower’s sign, and skeletal muscle biopsy revealed myopathic changes with core-like structures. Genomic investigation of this index patient lead to the discovery of a novel genetically defined CM subtype based on bi-allelic mutations in the uncoordinated mutant number-45 myosin chaperone B (*UNC45B*) gene. Regarding medical history, the mother reported reduced fetal movements during pregnancy. After birth, the patient presented as a floppy infant with feeding difficulties, improving after the first year of life. He was able to sit and walk independently at 10 and 20 months of age, respectively. Currently, his Gower’s time is >10s (Fig. 1b), he is unable to run, shows a Trendelenburg sign (Additional file 1: Figure S1h), he is overweight and has a static disease course. He talks with a nasal voice without chewing or swallowing difficulties. Facial weakness and ophthalmoplegia are absent. While he had a reduced forced vital capacity of 70%, his echocardiogram showed a normal heart function. Serum creatinine kinase levels were not elevated. For further clinical details, please see Additional file 2: Supplementary Material.

At the age of 10 years, left femoral quadriceps muscle biopsy showed myopathic changes, i.e., fiber size variability including hypertrophic and atrophic fibers and central nuclei (Fig. 1c) with core-like lesions mainly in the center of muscle fibers (Fig. 1d). Fiber type distribution was altered as type-2 fibers were virtually absent (Fig. 1d, Additional file 1: Figure S1a)**.** Small neonatal myosin positive fibers indicated regeneration (Additional file 1: Figure S1b). Electron microscopy unraveled numerous core-like alterations of myofibrillary architecture with Z-band streaming (Fig. 1f). Some mitochondria showed prominent matrix granula, globoidal inclusions, and even paracristalline intramitochondrial inclusions (Additional file 1: Figure S1f). We also observed subsarcolemmal accumulations of organelles (Additional file 1: Figure S1e).

To uncover underlying disease-causing mutations, we performed whole-exome sequencing (WES) (Additional file 2: Table S1 and Supplementary Material) [3]. By stringent filtering for various inheritance models (Additional file 2: Table S2), the most likely autosomal-recessive model in a consanguineous family let us to the solution. Based on skeletal muscle expression levels (Additional file 1: Figure S1 g) and previously reported animal models [4, 9, 11, 13, 21], we consider a strictly conserved homozygous base pair exchange in *UNC45B* (NM_173167:c.2261G > A, p.Arg754Gln, Fig. 1g-j) in a homozygous region as pathogenic. The variant leads to an amino acid substitution with a change in polarity and mass (dissimilarity 43 in Sneath’s index) in the armadillo repeat domain 3 (ARM3), and is reported with a CADD Score of 35. *UNC45B* is highly conserved and constrained against loss-of-function variants in the gnomAD population database (Additional file 2: Table S3). UNC-45 proteins show a three-domain configuration, with an N-terminal tetratricopeptide repeat (TPR) domain, poorly conserved central domain, and a C-terminal UCS domain (Unc45−/Cro1p−/She4p-related protein) [13]. Three consensus TPR repeats participate in protein-protein interaction especially with Hsp70 and Hsp90 [17]. The C-terminal UCS domain has been shown to form a putative myosin-binding groove, largely stabilized by electrostatic interactions [6]. Our patient’s missense mutation is located in the third ARM domain at residue p.Arg754 at the C-terminal UCS region, which might abrogate the interaction between UNC45B and myosin heavy chain [19], thus impairing myofibrillogenesis (Additional file 1: Figure S2d). Indeed, *in-silico* modeling and docking studies of the human UNC45B protein showed that the binding groove in the UCS domain is a negatively-charged surface at the R18R19 helices of UNC45B and serves as “place-holder” for the charged loop-U and β-sheets residues of myosin (MYH7) [6]. The p.Arg754Gln mutation is actually found directly in R18H1 [6, 12]. We calculated a change to a decreased isoelectric point and lower net charge from wildtype to p.Arg754Gln mutant in the R18-R19 residues at pH 7.4 by using the Prot-pi tool (Additional file 1: Figure S2c).

Notably, all three isoforms of UNC45B are highly expressed in skeletal muscle and the p.Arg754Gln mutation affects all isoforms (Additional file 1: Figure S2a,b), only one of the three isoforms is highly expressed in cardiac muscle. In a *D. melanogaster* model, an Unc-45 knockdown showed a severe cardiac phenotype with dilated cardiomyopathy and reduced muscle contractility [15]. *Unc-45b* knockdown in zebrafish and also the steif/unc-45b mutants resulted in paralysis and cardiac dysfunction based on severely disrupted myofibrillogenesis [4, 21]. Therefore, our patient might develop cardiomyopathy at later ages. Knockdown of *unc-45b* severely affected sarcomere organization including M- and Z-lines of skeletal muscles of embryos [2].

From the essential physiological function of UNC45B in muscle development, it can be deducted that deleterious genetic variants may lead to myopathy. On a multimeric level, UNC45B aggregates to a chain which serves as an assembly line for beta (β)-myosin heavy chain, encoded by *MYH7* [1, 5], and functions as a “template” defining the geometry, regularity, and periodicity of myosin arranged into muscle thick filaments [16]. After myosin incorporates into thick filaments, Unc45b and Hsp90 dissociate from myosin ensuring the proper myosin filament assembly [15]. Once disassociated, UNC45B binds to a VCP cofactor protein UFD-2 and an E3 ligase CHIP which leads to poly-ubiquitylation of UNC45B and its subsequent proteasomal degradation [10] (Additional file 1: Figure S2d).

Therefore, we hypothesize that our patient’s mutation in UNC45B in the UCS domain might directly lead to myofibrillogenesis failure (Additional file 1: Figure S2d). Of note, a heterozygous missense variant in *UNC45B* (c.2413C > T, p.Arg805Trp) has been reported to cause a dominant form of glaucoma without further confirmation since publication [8]. Noteworthy, there is a high heterozygous allele carrier status of 18/272310 in gnomAD of this c.2413C > T variant in healthy adults.

In conclusion, we here report for the first time a patient with an *UNC45B* mutation causing a novel genetically defined congenital myopathy disease entity. Our discovery is in line with the previously described myopathological phenotypes in *C. elegans* and in vertebrate mutants and knockdown–models [4, 7, 9, 11, 19].

## Supplementary information

**Additional file 1: Figure S1.** Further myopathological, electron microscopical and phenotypic findings in our patient with *UNC45B* variant. **Figure S2.** Gene and isoform expression of *UNC45B* in various tissues and a possible disease model scheme.

**Additional file 2: Table S1.** Detailed metrics of Whole Exome Sequencing in our patient with coverage (1x, 2x, 10x, 20x, 30x, 100x, mean). **Table S2.** Results of the variant filtering and the specific criteria we applied on the dataset. **Table S3.** Prediction of pathogenicity for our patient’s *UNC45B* variant via multiple scoring tools.

## Data Availability

The next generation sequencing datasets generated and analyzed during the current study are not publicly available because of patient confidentiality and since we do not have informed consent for that.
