# Supplementary material for: Bi-allelic mutations in uncoordinated mutant number-45 myosin chaperone B are a cause for congenital myopathy
Source: Acta Neuropathol Commun. 2019 Dec 18;7:211. doi: 10.1186/s40478-019-0869-1 (PMC6921565; doi:10.1186/s40478-019-0869-1)
Supplement: Supplementary file 1 — Additional file 1: Figure S1. Further myopathological, electron microscopical and phenotypic findings in our patient with UNC45B variant. Figure S2. Gene and isoform expression of UNC45B in various tissues and a possible disease model scheme. [file 40478_2019_869_MOESM1_ESM.zip › SUPPLEMENTARY FIGURE LEGENDS.docx]

**SUPPLEMENTARY FIGURE LEGENDS**

**Supplementary Fig. 1: Further myopathological, electron microscopical and phenotypic findings in our patient with *UNC45B* variant including gene expression in various tissues and a possible disease model. a** All muscle fibers correspond to type-1 fibers, whereas type-2 fibers were absent (insert: positive control). **b** Single regenerating atrophic muscle fibers scattered throughout the muscle (arrowheads). Immunohistochemistry with (a) mouse anti-myosin heavy chain fast component (clone MY-32; Biogenex, Fremont, CA, USA) and (b) mouse anti-myosin neonatal (clone WB-MHCn; Leica, Nussloch, Germany), slight counterstaining with hemalum. Original magnification x400 (a), x800 (b). **c** Core-like structure with prominent Z-band streaming in electron microscopy (EM). **d** Region of advanced myofibrillary disintegration in EM. **e** Abnormal subsarcolemmal accumulation of organelles in EM. **f** Mitochondria containing globoid and paracrystalline inclusions in EM. **g** Patient’s left lower limb and foot with contractures of the Achilles tendon. **h** Trendelenburg sign in our patient while walking.

**Supplementary Fig. 2: Gene and isoform expression of *UNC45B* in various tissues and a possible disease model scheme. a** Gene expression for *UNC45B* (ENSG00000141161.11) taken from GTex database portal sorted after median values. Highest expression in skeletal muscle, followed by heart (left ventricle and atrial appendage). Low expression in all other tissues, e.g., lung, peripheral nerves or brain. **b** Isoform expression of the three UNC45B isoforms in the top five tissues with highest read counts (skeletal muscle, heart left ventricle, heart atrial appendage, coronary artery, and testis, minimum shade white, maximum shaded dark blue, see legend). ENST00000394570.6 with 929 amino acids (aa; CCDS45648, NM_001033576.2, NP_001028748.1 and NM_001267052.2, NP_001253981.1) shows high expression in skeletal and cardiac muscle and mild expression in coronary artery. ENST00000591048.2 with 850 aa (CCDS76993, NM_001308281.1, NP_001295210.1) and ENST00000268876.9 with 931 aa (CCDS11292, NM_173167.3, NP_775259.1) show high expression in skeletal muscle and comparatively low to mild expressions in other tissues. The 850-aa isoform differs from the 931-aa isoform in that the amino acids 485-565 are missing (numbers based on 931-aa isoform), i.e. a small part of the central region and a longer part of the C-terminal UCS region (Unc45-/Cro1p-/She4p-related protein) that serves as a binding partner to myosin. Amino acids 564 and 565 (alanine and glycine, numbers based on 931-aa isoform) missing in 929-aa isoform which may cause different protein features, e.g., thermal stability or activity. Drawings of these three protein isoforms with changes of central and UCS region in each isoform created with IBS Biocuckoo [16], each with 3 N-terminal tetratricopeptide repeats (TPR), central regionand C-terminal UCS region, and 3 armadillo repeat domains (ARM) domains below. **c** Titration curves based on Prot pi calculation of isoelectric point and net charge at pH 7.4 reveal decrease in isoelectric point from healthy R18R19 myosin-binding groove pI=6.023 to our patient’s p.Arg754Gln mutation pI=5.015, as well as a lower net charge at pH 7.4from z=-0.786 to z=-1.786, respectively (UNC45B NP_775259.1 SDKLRQKIFKERALPDIENYMFE and the p.Arg754Gln mutation from our patient SDKLQQKIFKERALPDIENYMFE) (https://www.protpi.ch/Calculator/ProteinTool). **d** Possible pathophysiological model for identified *UNC45B* variant in our patient. In steady state conditions, myosin associates with Hsp90 and normal UNC45B to ensure myosin head folding (A). While UNC45B disassociates from folded myosins and forms another complex with UFD2/CHIP to become poly-ubiquitylated and degraded by proteasomes (B), folded myosins develop proper thick filaments (C). In pathological conditions, a mutation in *UNC45B* causes a decreased interaction with Hsp90 and myosin. An elevated interaction with myosin leads to a failure in myofibrillogenesis and increase in proteasomal degradation of myosin. Additionally, a mutation in *UNC45B* might alter its cellular expression pattern by interfering in its interaction with ubiquitin-proteasome system components. Human protein nomenclature used in illustration.
