# Supplementary figures and images for: Bi-allelic mutations in uncoordinated mutant number-45 myosin chaperone B are a cause for congenital myopathy
Source: Acta Neuropathol Commun. 2019 Dec 18;7:211. doi: 10.1186/s40478-019-0869-1 (PMC6921565; doi:10.1186/s40478-019-0869-1)

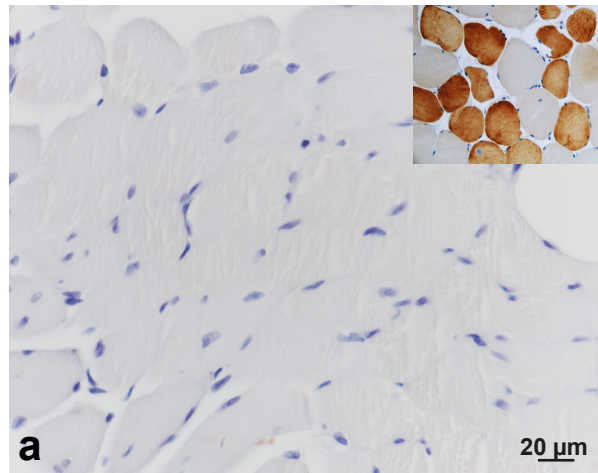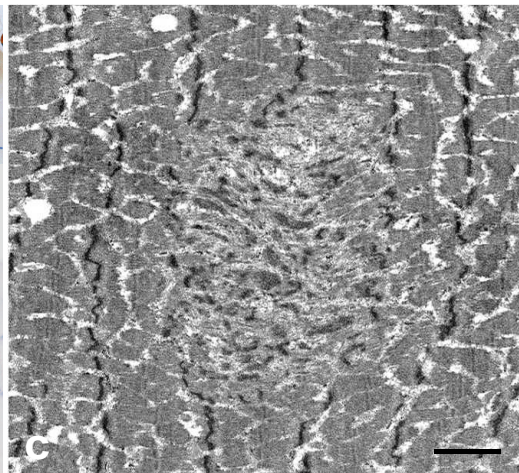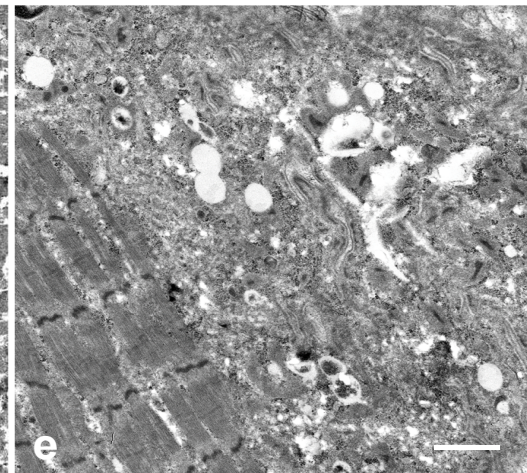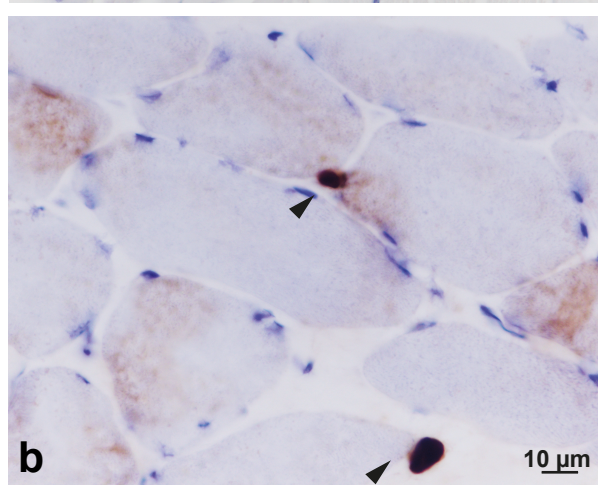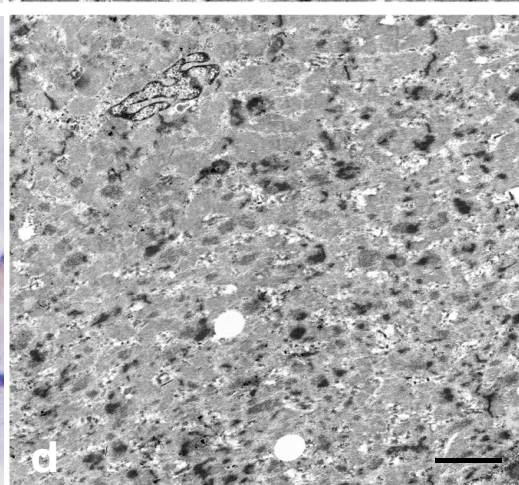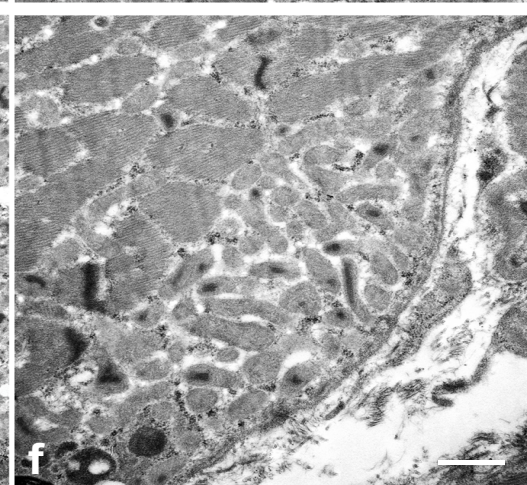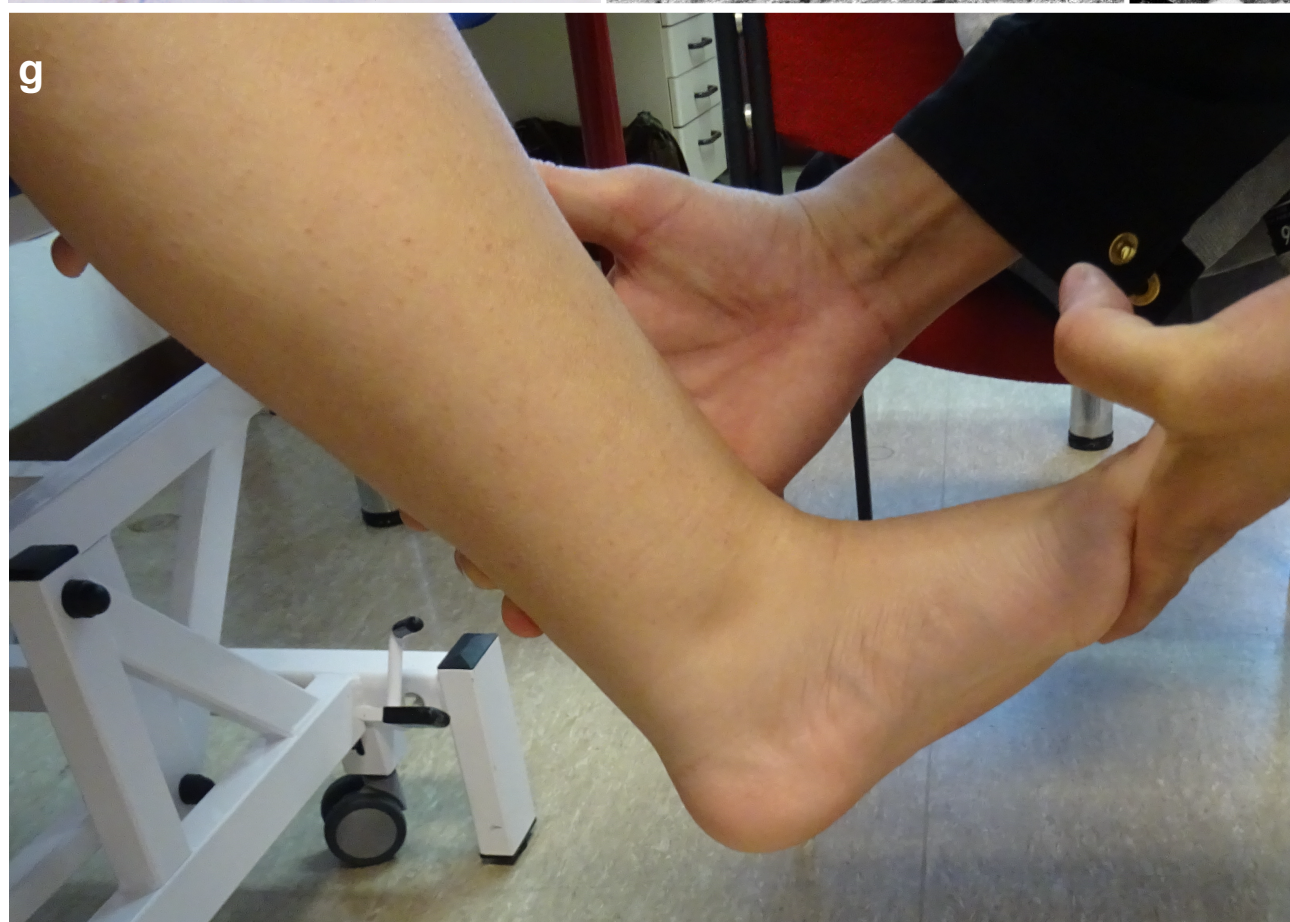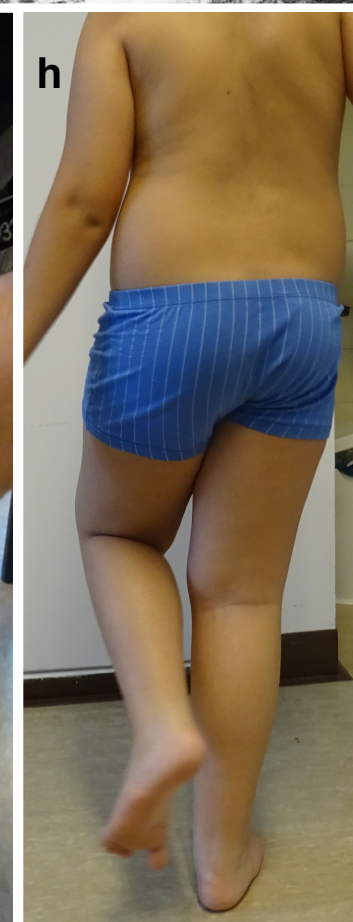

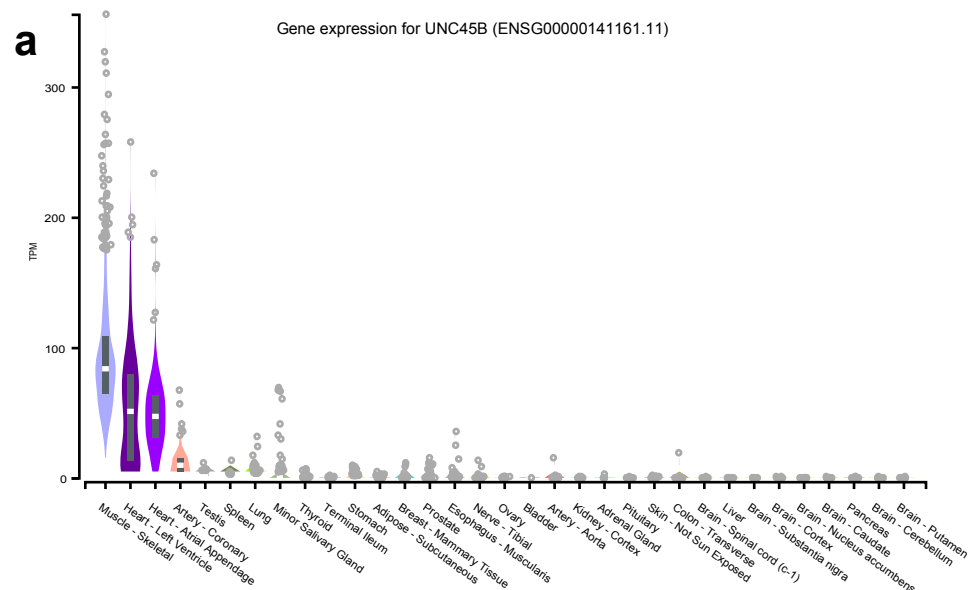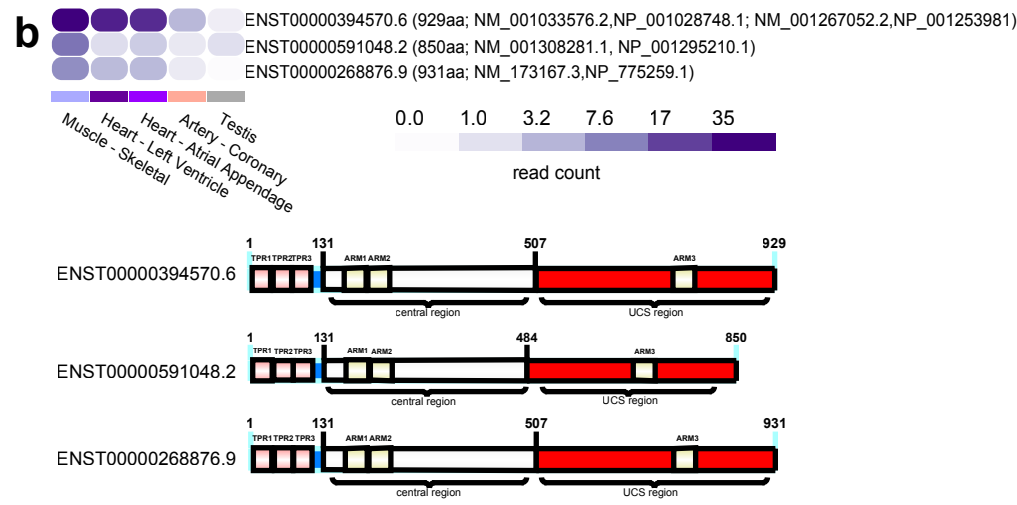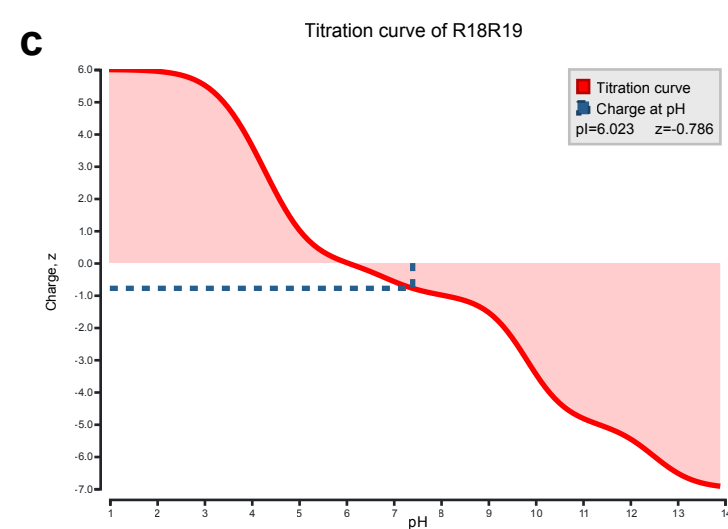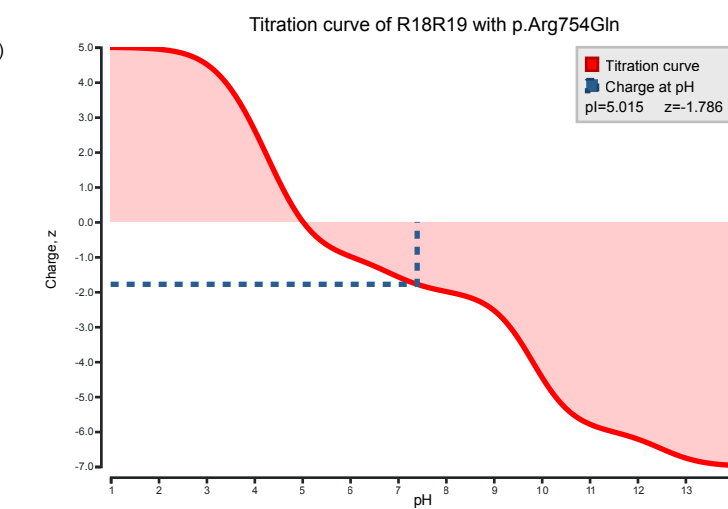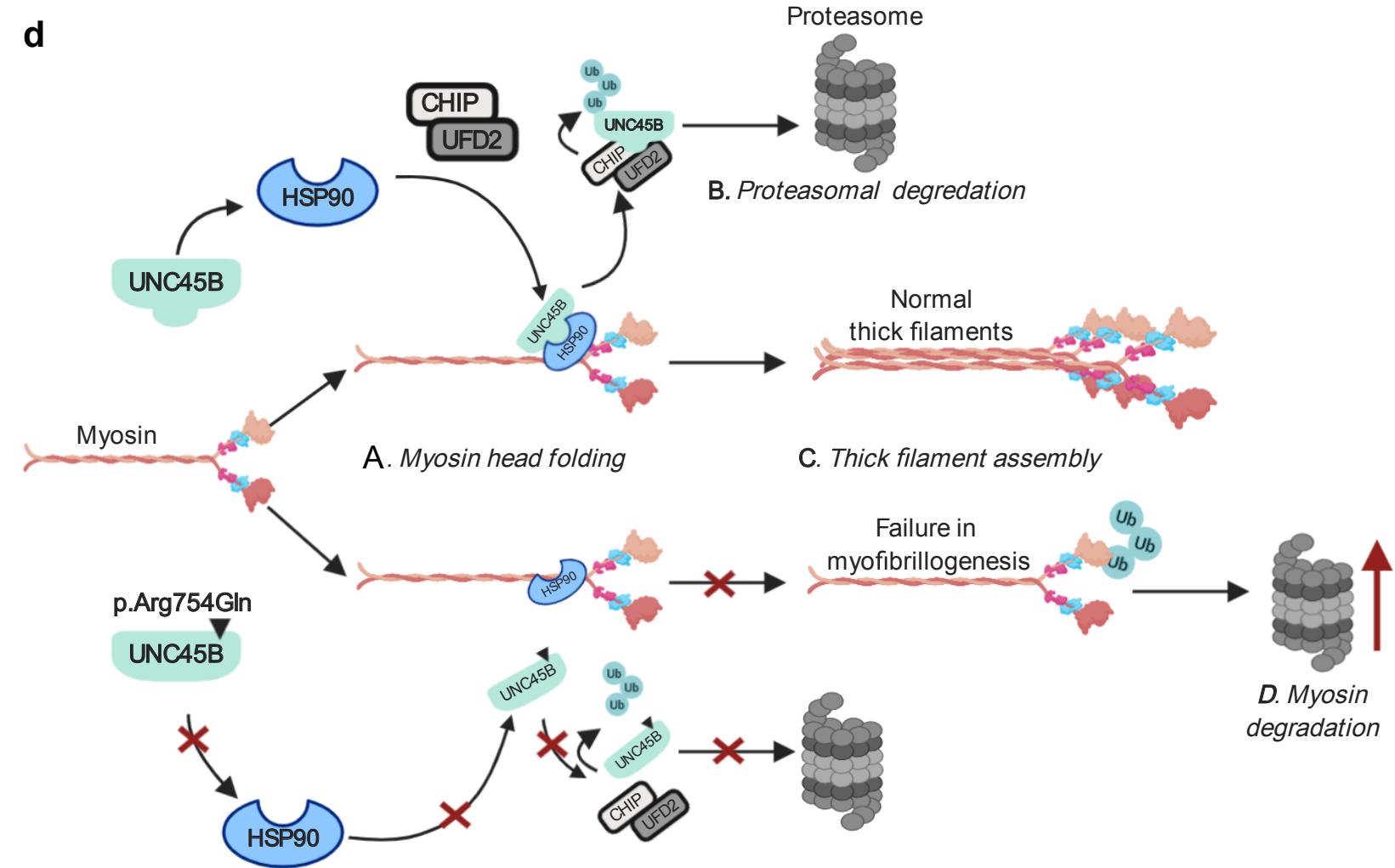

Supplement: Supplementary file 1 — Additional file 1: Figure S1. Further myopathological, electron microscopical and phenotypic findings in our patient with UNC45B variant. Figure S2. Gene and isoform expression of UNC45B in various tissues and a possible disease model scheme. [file 40478_2019_869_MOESM1_ESM.zip › SupplementaryFigures.pdf]
