# Supplementary material for: Bi-allelic mutations in uncoordinated mutant number-45 myosin chaperone B are a cause for congenital myopathy
Source: Acta Neuropathol Commun. 2019 Dec 18;7:211. doi: 10.1186/s40478-019-0869-1 (PMC6921565; doi:10.1186/s40478-019-0869-1)
Supplement: Supplementary file 2 — Additional file 2: Table S1. Detailed metrics of Whole Exome Sequencing in our patient with coverage (1x, 2x, 10x, 20x, 30x, 100x, mean). Table S2. Results of the variant filtering and the specific criteria we applied on the dataset. Table S3. Prediction of pathogenicity for our patient’s UNC45B variant via multiple scoring tools. [file 40478_2019_869_MOESM2_ESM.docx]

**SUPPLEMENTARY MATERIAL**

**Article title: Bi-allelic mutations in uncoordinated mutant number-45 myosin chaperone B are a cause for congenital myopathy**

Journal name: Acta Neuropathologica

Author names and affiliations:

Hormos Salimi Dafsari^1,2^, Nur Mehpare Kocaturk^1,2^, Hülya-Sevcan Daimagüler^1,2^, Anna Brunn^3^, Jörg Dötsch^1,4^, Joachim Weis^5^, Martina Deckert^3^, Sebahattin Cirak^1,2,4^

1 Department of Pediatrics, Faculty of Medicine and University Hospital Cologne, University of Cologne, Germany

2 Center for Molecular Medicine (CMMC), Faculty of Medicine and University Hospital Cologne, University of Cologne, Germany

3 Institute of Neuropathology, Faculty of Medicine and University Hospital Cologne, University of Cologne, Germany

4 Center for Rare Diseases, Faculty of Medicine and University Hospital Cologne, University of Cologne, Germany

5 Institute of Neuropathology, RWTH University Hospital, Aachen, Germany

Corresponding author: Dr. med. Dipl. Chem. Sebahattin Cirak, University Hospital Cologne, Department of Pediatrics, E-mail: sebahattin.cirak@uk-koeln.de

**CLINICAL PHENOTYPE**

For this study, the patient was recruited to our neuromuscular clinic and center for rare diseases. We obtained written informed consent from the patient’s parents. The study was approved by the ethics committee of the Medical Faculty, University Hospital Cologne, University of Cologne (17-096).

The now 11-year old male patient was referred to our neuromuscular clinic due to proximal muscle weakness. From medical history, the mother reports that the pregnancy was complicated due to weak fetal movements in utero. The patient was born at term with Caesarian section in breech presentation with an initially unremarkable postnatal cardiorespiratory adaptation. He presented as a floppy infant with feeding difficulties. Gastrointestinal reflux repeatedly led to episodes of apnoea and pneumonia. Throughout the first year of life, recurrent pneumonia led to repeated hospitalization and systemic antibiotic treatment. After a partial Thal-fundoplication (270° anterior) at 7 months of age, the patient’s reflux ceased, and the patient started to thrive better. Clinically, he had as an infant a plagiocephaly, high-arched palate, anosacral naevus, cryporchism, and a funnel chest. Physiotherapy was started after the first month of life. The patient’s muscle weakness improved after the first year of life. His motor milestones were delayed with sitting at 10 months and independent walking at 20 months of age. At the most recent out-patient control in our clinic, he showed a rather static disease course with a Gower´s time of >10sec, a Trendelenburg sign, and a bilateral Achilles tendon contractures with a -30°flexion deficit (**Fig. 1a,b** and **Supplementary Fig. 1g,h**). He was not able to run; however, the walking distance was not limited in daily life with a waddling gait. Furthermore, he talked with a nasal voice, but he did not show chewing or swallowing difficulties. His intellectual and speech development were age appropriate. The patient receives good grades at secondary school. The patient’s family is of Lebanese-Arabic descent without any history of neurological diseases.

The newborn screening according to German national standards [24] as well as specific neurometabolic and endocrinological examinations were unremarkable (CDG, beta-oxidation defects, cortisol, parathormone, DHEA-S, IGF-1, 25-OH vitamin D, TSH, fT3, fT4). At the age of 11 years, we observed a mild microcytic hypochromic anemia (Hb 10.4g/dl, ref. 11.5-15.0; MCV 65fl, ref. 80-96; MCH 20pg, ref. 23-24). Of note, CK levels (26 IU/l at 4 months, 29 IU/l at 10 years) as well as autoantibody investigations for skeletal muscle, acetylcholine, and titin were unremarkable.

Echocardiography and electrocardiography showed no hints of a cardiac involvement. An electroencephalography at the age of 4 months was unremarkable. At the age of 10 years, the patient showed a restrictive lung disease with a forced vital capacity (FVC) of 59% (normal range >80%) and at 11 years of age an FVC of 70%. An X-ray investigation of the left hand showed a bone age of 4 months at an actual age of 10 months. A cranial MRI scan at the age of 4 months war normal. Head circumference was at 51.2cm at the age of 10 years (3. percentile, z-score -1.82) and 51.5cm at the age of 11 years (3. percentile, z-score -1.88). At the age of 4 months, somatosensory evoked potentials in tibialis and medianus nerves were unremarkable.

Diagnostic testing by targeted dideoxy sequencing for known mutations in coding exons of *GAA*, *DOK7*, *CHRNE*, and *SEPN1,* as well as copy number testing for *SMN1* by MLPA and methylation testing for Prader-Willi syndrome, did not show any remarkable findings.

**HISTOPATHOLOGICAL METHODS**

For morphological analysis, a biopsy was obtained from the patient’s left femoral quadriceps muscle (14 x 10 x 4mm) and snap-frozen in isopentane (Fluka, Neu-Ulm, Germany), precooled in liquid N2 prior to preparation of 9 µm-thick serial frozen sections used for histochemistry, enzyme histochemistry, and immunohistochemistry. Immunohistochemistry was performed with mouse anti-myosin-heavy chain fast component (clone MY-32; Biogenex, Fremont, CA, USA) and mouse anti-myosin neonatal (clone WB-MHCn; Leica, Nussloch, Germany). Glutaraldehyde-fixed, epon-embedded muscle specimen was used for electron microscopy (EM, **Fig. 1f** and **Supplementary Fig. 1c-f**) [31].

**HISTOPATHOLOGICAL RESULTS**

Core-like structures were detected mainly in the center of muscle fibers (arrowheads in **Fig. 1d**) in NADH histochemistry. Spectrin, dys1, dys2, dys3, desmin, and alpha-actinin were regularly expressed.

Immunohistochemistry with anti-myosin-heavy chain fast component revealed a lack of type-2 fibers (**Supplementary Fig. 1a**); regenerating atrophic muscle fibers were evidenced by immunostaining with mouse anti-myosin neonatal (**Supplementary Fig. 1b**). Focally, myofibrillar disintegration was obvious (arrows in **Fig. 1e**).

Electron microscopy showed numerous focal core-like alterations (**Supplementary Fig. 1c)** of myofibrillar architecture with Z-bands streaming, less frequently, regions of more pronounced myofibrillar disintegration (**Supplementary Fig. 1d**). We further observed very few, small membranous plasmatic bodies. Some mitochondria show prominent matrix granula, globoidal inclusions, and even paracristalline intramitochondrial inclusions (**Supplementary Fig. 1f**). We also observed subsarcolemmal accumulations of organelles (**Supplementary Fig. 1e**).

**GENOMIC INVESTIGATION**

To uncover the underlying disease-causing gene, whole exome sequencing (WES) was performed on an Illumina NovaSeq 6000 Sequencer (Illumina, USA) with a paired-end 100 bp sequencing protocol according to the manufacturer’s best-practice protocol, after enrichment with the SureSelect Human All Exon V.6 (Agilent, USA) kit with a mean coverage of 95 fold. Please see **Supplementary Table 1 (Online Resource 1)** for detailed metrics of WES.

**Table S1**

| **Cov 1x** | **Cov 2x** | **Cov 10x** | **Cov 20x** | **Cov 30x** | **Cov 100x** | **Mean Cov** | **Total reads** | **ROH_Sum** |
| --- | --- | --- | --- | --- | --- | --- | --- | --- |
| 98.3 | 98.1 | 97.2 | 94.2 | 87.9 | 33.0 | 95 | 126011250 | 298 |

**Table S1**: Detailed metrics of Whole Exome Sequencing in our patient with coverage (1x, 2x, 10x, 20x, 30x, 100x, mean), number of total reads and summary of autosomal Runs of Homozygosity (ROH) in megabases (ROH_Sum) of 298 based on ALLEGRO [7]. The following parameter settings were used to extract high quality SNVs for the ROH calculation: Maximal observed allele frequency <=0.8% in any gnomAD population for ROH calculation; Genotype Quality >=99, as Phred-scaled confidence that genotype assignment is correct, derived from Phred-scaled likelihood of the considered genotypes in the variant record for each sample; Log odds of being a true variant versus being false under the trained Gaussian mixture model >=2 (with GATK according to Mckenna et al [18]); Quality by Depth>=6; Mapping Quality>=60; Phred-scaled p-value using Fishers exact test to detect strand bias<=20; Z-score from Wilcoxon rank sum test of Alternate vs. Reference Alleles read mapping qualities>=-2.5; Rank Sum Test for relative positioning of Reference versus Alternate alleles within reads >=-2.5

The annotated variant lists were uploaded to the Cologne Center for Genomics Varbank 2 (https://varbank.ccg.uni-koeln.de/varbank2) database for variant filtering which was previously published [20]. The main differences between Varbank versions 1 and 2 are major redesign updates on hardware and software components, e.g., its usage of GRCh38 and ENSEML b90, detailed copy number variant detection, combining and functional annotation with support for gnomAD, ClinVar, and LCR-filter lists. We performed variant filtering for rare pathogenic variants below an allele frequency of 0.1% with autosomal recessive consanguine, compound recessive, and dominant inheritance models. Further, we assumed that homozygous variants or deletions were likely embedded in Runs of Homozygosity (see **Supplementary Table 1** for settings of ROH) due to the consanguinity of parents, and looked for variations in the ROH, but also considered other theoretically possible inheritance patterns.

The evaluation of the pathogenicity of variants was performed with the use of scripted tools to evaluate the effects on gene and protein level (see below) and utilizing data from various public databases to see the distribution of genetic variants in healthy probands: dbSNP, [26]; ENSEMBL, [6]; commercial HGMD professional database, [28] and ClinVar (www.ncbi.nlm.nih.gov/clinvar/). We further used a collection of tools summarized as *MedPred* score which contain the following pathogenicity prediction scorings from dbNSFP (https://sites.google.com/site/jpopgen/dbNSFP): POLYPHEN2_HDIV and _HVAR, [1], SIFT [13], RVIS [19], LRT, MutationTaster, Mutation Assessor, FATHMM, PROVEAN, VEST3, MetaSVM, MetaLR, M-CAP, REVEL, MutPred 1.2, CADD, DANN, FATHMM-MKL, Eigen-PC, GenoCanyon, fitCons_integr, fitCons_GM12878, fitCons_H1-hESC, fitCons_HUVEC, Gerp++_RS [3], phyloP100way_vert, phyloP20way_mamm, phastCons100way_vert, phastCons20way_mamm, SiPhy_29way, ADA_score, and RF_score. A splice site analysis was performed, based on Yeo and Burge, 2004 [32]. As this was only available for the U2-type introns, a second position weight matrix model [27] was applied for the U2/U12 core splice sites and in addition for the U2/U12 branch points. The functional interpretation identified the most probable intron types. Filtering was then based on the score change in percent.

In the compound heterozygous model, we were left with no rare functional variants (RFVs) with the above-mentioned filtering criteria that classified as non-synonymous coding or indels, including intronic mutations close to splice site <20bp up-/downstream. In the autosomal dominant model, four RFVs remained and two of these were predicted as non-polymorphisms with a MedPred score above 0.5. However, these RFVs were in genes (*NCKAP5,* *GTSE1* and *TREH*) that were not associated with previously published functional data in neuromuscular models, muscle-specific tissue expression nor phenotype in a neuromuscular disease, neither any other Mendelian disease. However, our patient merely showed a muscle-specific phenotype without involvement of any other organs. The gnomAD population database showed an allele count (AC) of 37/143142 for the *TREH* variant. The GTSE1 variant was reported with an AC of 1/143340 in gnomAD and 2/125568 in TOPDMED database. Thus, both variants were observed in a healthy adult population and could be excluded in an autosomal dominant inheritance model with high penetrance leading to severe muscle weakness. We were not able to fully exclude the *NCKAP5* variants based on data from population databases but *NCKAP5* variant was predicted as a polymorphism with low functional effect with a MedPred score of 0.10. Thus, we excluded *NCKAP5, GTSE1,* and *TREH* as disease-causing variants.

In the autosomal recessive consanguine model, we were left with 12 RFVs. Nine variants were predicted to have a functional impact (MedPred score >0.5) and only 3 RFVs were previously associated with a muscle-specific functional model or tissue expression (*UNC45B, EEA1, KANK1*, **Supplementary Table 2**).

We analyzed for co-segregation with the disease phenotype by dideoxy sequencing, i.e., patient’s mother and three sisters. There was no co-segregation with the disease phenotype in *KANK1*, but for both *UNC45B* and *EEA1* variants there was co-segregation with the disease phenotype in the family. Based on gene expression derived from the GTex database (**Supplementary Fig. 2a**) and previously published reports from *C. elegans* and *steif* *D. rerio* knockout models with paralysis and defective myofibril organization [2,30,8,14], we consider a strictly conserved homozygous base pair exchange in *UNC45B* (NM_173167:c.2261G>A, p.Arg754Gln, **Fig. 1g,i,j**) in a homozygous linkage region to be the likely pathogenic disease-causing variant. We had no strong evidence for the RFV in *EEA1* based on missing muscle-specific literature and comparatively low skeletal muscle expression levels from GTex database (www.gtexportal.org; *UNC45B* in **Supplementary Fig. 2a,b** ; *EEA1* not shown). An EEA1 deficiency would result in decreased or smaller vesicles as well as an inefficient fusion of internalized vesicles into early endosomes during endocytosis [21]. An EEA1 deficiency would be expected to lead to an early endosomal defect in cells and tissues ubiquitously. Disorders with an early endosomal defect present as multisystem disorder with neurodevelopmental or neurodegeneration, e.g., Niemann-Pick Type C or Huntington’s disease [25,12]. Our patient merely showed an exclusive neuromuscular disorder. However, the *EEA1* variant might also have a disease-modifying effect. Thus, although we could not fully exclude the variant in *EEA1*, it seems unlikely for the variant to be the primary disease-causing variant for our case for a myopathy-only phenotype.

**Table S2:**

| ROH:298 | Autosomal recessive consanguineous | Autosomal dominant | Compound heterozygous |
| --- | --- | --- | --- |
| Rare functional variants (RFV) | 12 | 3 | 0 |
| Non-polymorphism predictions (MedPred) | 9 | 2 | 0 |
| RFVs in muscle-specific gene (function, expression, phenotype) | 3 | 0 | 0 |
| RFVs and gene constraint score | **1** | 0 | 0 |

**Table S2:** Results of the variant filtering and the specific criteria we applied on the dataset. With a Runs of Homozygosity (ROH) of 298, we first applied the autosomal recessive consanguineous filtering model. We filtered out 12 rare functional variants (RFV), out of which nine variants were predicted with substantial functional impact (MedPred score >0.5). Only three variants were associated with a muscle-specific functional model, tissue expression or disease phenotype: *UNC45B* with MedPred score 0.75*, EEA1* with MedPred score 0.60*, KANK1* with MedPred score 0.78. Dideoxy sequencing showed co-segregation with the disease phenotype in *UNC45B* and *EEA1,* while *KANK1* did not and was formally ruled out. The gene constraint scores for both *UNC45B* and *EEA1* suggested that these genes are constrained against loss-of-function variants (**Supplementary Table 3**). However, based on functional animal models we consider a strictly conserved homozygous base pair exchange in *UNC45B* (NM_173167:c.2261G>A, p.Arg754Gln, **Fig. 1g,i,j**) in a homozygous linkage region to be pathogenic. We employed an autosomal dominant filtering scheme with a maximum gnomAD allele frequency of 0.1% and filtered out 3 RFVs (*NCKAP5*, *GTSE1*, and *TREH*). Only two variants survived filtering for functional impact by multiple variant pathogenicity predictions (*GTSE1*, *TREH*), however none of these two variants were absent in the gnomAD population database and are unlikely to cause a rare and severe disease as a de-novo variant. Also, none of these variants were in genes with an associated phenotype, with previous functional analyses that may have fitted our muscle-specific phenotype, nor did these genes show particularly high gene constraint scores. We applied a compound heterozygous filtering scheme and no RFVs were filtered out.

Multiple sequence alignment of UNC45B indicates that the residue p.Arg754 is strictly conserved throughout species (NP_775259.1 homo sapiens, mutated sequence from our patient c.2261G>A p.Arg754Gln, XP_001174363.2 p. troglodytes, XP_0011113905.2 m. mulatta, XP_005624856.1 c. lupus, XP_002695676.1 b. taurus, NP_848795.3 m. musculus, NP_001100498.1 r. norvegicus, XP_004946569.1 g. gallus, NP_705959.1 d. rerio, NP_001172057.1 x. tropicalis, NP_524796.1 d. melanogaster, XP_310258.5 a. gambiae, and NP_497205.1 c. elegans, **Fig. 1h**). For the alignment, we used the NCBI HomoloGene Protein Multiple Alignment platform and downloaded the protein sequences in FASTA format (0bp up- and downstream of gene), the multiple sequence alignment was generated by the MUSCLE algorithm version 3.6 (using option: -maxiters 2). [4] For visualization, we used the Jalview online tool (version 2.11.0) [29] (**Fig. 1h**). Amino acid color labels were selected for the block substitution matrix 62 (BLOSUM62).

We further obtained information on gene tissue and isoform expression (ENSG00000141161.11) taken from GTex database portal sorted after median values. We looked at the three UNC45B isoforms in the top five tissues with highest read counts, i.e., skeletal muscle, heart left ventricle, heart atrial appendage, coronary artery and testis: ENST00000394570.6 with 929 amino acids (CCDS45648, NM_001033576.2, NP_001028748.1 and NM_001267052.2, NP_001253981.1), ENST00000591048.2 with 850 amino acids (CCDS76993, NM_001308281.1, NP_001295210.1), and ENST00000268876.9 with 931 amino acids (CCDS11292, NM_173167.3, NP_775259.1), tissue and isoform expression data in **Supplementary Fig. 2a**, protein isoforms with domains shown in **Supplementary Fig. 2b**. In comparison, Unc45b transcripts in mice only show a 929-aa and a 931-aa long isoform, while zebrafish only show a 934-aa long unc45b isoform. The 850-aa isoform differs from the 931-aa isoform in that the amino acids 485-565 are missing (numbers according to 931-aa isoform, i.e. a part of the poorly conserved central region and also of the C-terminal UCS region (Unc45-/Cro1p-/She4p-related protein) that serves as a binding partner to myosin. The 929-aa isoform only has amino acids 564 and 565 (alanine and glycine, numbers according to 931-aa isoform) missing in the UCS region when compared to 931-aa isoform which may cause different protein features, e.g., thermal stability or activity. Further research must be done on the isoforms on protein level to improve the understanding of tissue-specific function and expression.

For understanding the electrostatic changes in the mutated part of the protein due to the p.Arg754Gln substitution, we performed a simulated calculation based on physico-chemical parameters of the amino acid residues in the putative myosin-binding groove R18-R19. Using the Prot-pi protein tool from the Center for Biochemistry and Bioanalytics at Zurich University of Applied Sciences (release 2.2.22.140, https://www.protpi.ch/Calculator/ProteinTool), we calculated a change in the isoelectric point (physiological pI=6.023 to mutation pI=5.015) and the net charge at pH 7.4 (z=-0.786 to z=-1.786, respectively) between the R18-R19 residues from UNC45B NP_775259.1 (SDKLRQKIFKERALPDIENYMFE, based on information from primary amino acid sequence and secondary structure from X-ray crystal structure examinations [15]) and the accordingly changed R18-R19 residues with the p.Arg754Gln missense mutation from our patient (SDKLQQKIFKERALPDIENYMFE).

**Table S3:**

| **Chr: Region (build** **GRCh38/hg38)** | **Exon** | **RefSeq**  NM_173167 | **GERP++** | **SIFT66** | **PolyPhen2** | **REVEL** | **Mutation-Taster2** | **CADD PHRED v1.3** | **MaxEntScan** | **dbscSNV** | **dbSNP** | **population databases** | **Gene constraint scores** |
| --- | --- | --- | --- | --- | --- | --- | --- | --- | --- | --- | --- | --- | --- |
| 17:35177610 | 17/20 | Homozygous. c.2261G>A, p.Arg754Gln | 5.3 | 0 | 0.554 | 0.509 | 1 | 35 | Alt: 8.281  Ref: 10.898  Diff: 2.617 | ADA: 0.999  RF: 0.968 | rs139715157, not reported in ClinVar | gnomAD: Allele count 9/190556; no homozygotes | LoFTool: 0.84;  SORVA: 0;  P(HI): 0.218; P(rec): 0.109; GDI-Phred: 7.209; RVIS: 71.68 perc.; RVIS (ExAC-0.05%): 92 perc. |
|  |  |  |  |  |  |  |  |  |  |  |  | GME: no report |  |
|  |  |  |  |  |  |  |  |  |  |  |  | Iranome: no report |  |

**Table S3: Prediction of pathogenicity for our patient’s *UNC45B* variant via multiple scoring tools.** GERP++: Genomic Evolutionary Rate Profiling rejected substitutions score, range from minimum -12.3 to maximum 6.17 [3]; SIFT: Sorting Intolerant from Tolerant Substitutions score, version ensemble 66, range from 0.00 (deleterious) to 1.00 (tolerated) [13]; PolyPhen2: Polymorphism Phenotyping-2 score, range from minimum 0.00 (benign) to maximum 1.00 (damaging) [1]; REVEL: Rare exome variant ensemble learner score for predicting the pathogenicity of rare missense variants, range from minimum 0.00 to maximum 1.00 [10]; MutationTaster2: MutationTaster score, version 2, range from minimum 0.00 to maximum 1.00 (http://www.mutationtaster.org/); CADD-Phred v1.3: Combined Annotation-Dependent Depletion scores and [23], Phred-like score with minimum 0.00 and maximum 40.00, version 3; MaxEntScan: prediction of potential splice site alteration (differential score “diff” close to commonly used threshold of 2) [32]; dbscSNV: prediction of splice site alteration, ADAscore and RF score both range minimum 0.00 to maximum 1.00 [17]; dbSNP: reports in Database of Single Nucleotide Polymorphisms 147 (hg38) (https://www.ncbi.nlm.nih.gov/snp/), no reports in ClinVar database (hg38) (https://www.ncbi.nlm.nih.gov/clinvar/); gnomAD population reports: Allele count and number of homozygotes in genome aggregation database (https://gnomad.broadinstitute.org/), no reports in Greater Middle Eastern Database (http://igm.ucsd.edu/gme/data-browser.php); Gene constraint scores: Loss of function tool (LoFTool) score, range from minimum 0.00 to maximum 1.00, indicate that the gene was in highest quartile and especially intolerant towards loss-of-function mutations [5], SORVA (Significance of rare variants) indicated that were no individuals in the 1000 Genomes Project data (N=2504) who were either Heterozygote or Homozygote of LOF SNVs with an MAF below 0.005, i.e., SORVA is a method for ranking genes based on mutational burden [22], P(HI) showed a rather low probability of a deletion causing a haploinsufficiency phenotype in this gene [9], P(rec) showed a low probability to be intolerant of homozygous LoF variants, GDI-Phred (a Phred-scaled gene damage index) showed low nonsynonymous mutational load in protein-coding gene in the general population [11], RVIS (Residual Variation Intolerance Score) showed that this gene is on the 71^st^ percentile (perc.) of genes with functional intolerance towards genetic variation than expected based on the apparently neutral variation found in the gene, also on 92^nd^ percentile in relation to ExAC population database with an MAF below 0.05%, thus neither RVIS scores support high functional intolerance [19].

**SUPPLEMENTARY FIGURE LEGENDS**

**Figure S1: Further myopathological, electron microscopical and phenotypic findings in our patient with *UNC45B* variant including gene expression in various tissues and a possible disease model. a** All muscle fibers correspond to type-1 fibers, whereas type-2 fibers were absent (insert: positive control). **b** Single regenerating atrophic muscle fibers scattered throughout the muscle (arrowheads). Immunohistochemistry with (a) mouse anti-myosin heavy chain fast component (clone MY-32; Biogenex, Fremont, CA, USA) and (b) mouse anti-myosin neonatal (clone WB-MHCn; Leica, Nussloch, Germany), slight counterstaining with hemalum. Original magnification x400 (a), x800 (b). **c** Core-like structure with prominent Z-band streaming in electron microscopy (EM). **d** Region of advanced myofibrillary disintegration in EM. **e** Abnormal subsarcolemmal accumulation of organelles in EM. **f** Mitochondria containing globoid and paracrystalline inclusions in EM. **g** Patient’s left lower limb and foot with contractures of the Achilles tendon. **h** Trendelenburg sign in our patient while walking.

**Figure S2: Gene and isoform expression of *UNC45B* in various tissues and a possible disease model scheme. a** Gene expression for *UNC45B* (ENSG00000141161.11) taken from GTex database portal sorted after median values. Highest expression in skeletal muscle, followed by heart (left ventricle and atrial appendage). Low expression in all other tissues, e.g., lung, peripheral nerves or brain. **b** Isoform expression of the three UNC45B isoforms in the top five tissues with highest read counts (skeletal muscle, heart left ventricle, heart atrial appendage, coronary artery, and testis, minimum shade white, maximum shaded dark blue, see legend). ENST00000394570.6 with 929 amino acids (aa; CCDS45648, NM_001033576.2, NP_001028748.1 and NM_001267052.2, NP_001253981.1) shows high expression in skeletal and cardiac muscle and mild expression in coronary artery. ENST00000591048.2 with 850 aa (CCDS76993, NM_001308281.1, NP_001295210.1) and ENST00000268876.9 with 931 aa (CCDS11292, NM_173167.3, NP_775259.1) show high expression in skeletal muscle and comparatively low to mild expressions in other tissues. The 850-aa isoform differs from the 931-aa isoform in that the amino acids 485-565 are missing (numbers based on 931-aa isoform), i.e. a small part of the central region and a longer part of the C-terminal UCS region (Unc45-/Cro1p-/She4p-related protein) that serves as a binding partner to myosin. Amino acids 564 and 565 (alanine and glycine, numbers based on 931-aa isoform) missing in 929-aa isoform which may cause different protein features, e.g., thermal stability or activity. Drawings of these three protein isoforms with changes of central and UCS region in each isoform created with IBS Biocuckoo [16], each with 3 N-terminal tetratricopeptide repeats (TPR), central regionand C-terminal UCS region, and 3 armadillo repeat domains (ARM) domains below. **c** Titration curves based on Prot pi calculation of isoelectric point and net charge at pH 7.4 reveal decrease in isoelectric point from healthy R18R19 myosin-binding groove pI=6.023 to our patient’s p.Arg754Gln mutation pI=5.015, as well as a lower net charge at pH 7.4from z=-0.786 to z=-1.786, respectively (UNC45B NP_775259.1 SDKLRQKIFKERALPDIENYMFE and the p.Arg754Gln mutation from our patient SDKLQQKIFKERALPDIENYMFE) (https://www.protpi.ch/Calculator/ProteinTool). **d** Possible pathophysiological model for identified *UNC45B* variant in our patient. In steady state conditions, myosin associates with Hsp90 and normal UNC45B to ensure myosin head folding (A). While UNC45B disassociates from folded myosins and forms another complex with UFD2/CHIP to become poly-ubiquitylated and degraded by proteasomes (B), folded myosins develop proper thick filaments (C). In pathological conditions, a mutation in *UNC45B* causes a decreased interaction with Hsp90 and myosin. An elevated interaction with myosin leads to a failure in myofibrillogenesis and increase in proteasomal degradation of myosin. Additionally, a mutation in *UNC45B* might alter its cellular expression pattern by interfering in its interaction with ubiquitin-proteasome system components. Human protein nomenclature used in illustration.

**REFERENCES**

1. Adzhubei IA, Schmidt S, Peshkin L, Ramensky VE, Gerasimova A, Bork P, Kondrashov AS, Sunyaev SR (2010) A method and server for predicting damaging missense mutations. Nature methods 7:248-249. doi:10.1038/nmeth0410-248

2. Bernick EP, Zhang PJ, Du S (2010) Knockdown and overexpression of Unc-45b result in defective myofibril organization in skeletal muscles of zebrafish embryos. BMC cell biology 11:70. doi:10.1186/1471-2121-11-70

3. Davydov EV, Goode DL, Sirota M, Cooper GM, Sidow A, Batzoglou S (2010) Identifying a high fraction of the human genome to be under selective constraint using GERP++. PLoS computational biology 6:e1001025. doi:10.1371/journal.pcbi.1001025

4. Edgar RC (2004) MUSCLE: multiple sequence alignment with high accuracy and high throughput. Nucleic acids research 32:1792-1797. doi:10.1093/nar/gkh340

5. Fadista J, Oskolkov N, Hansson O, Groop L (2017) LoFtool: a gene intolerance score based on loss-of-function variants in 60 706 individuals. Bioinformatics (Oxford, England) 33:471-474. doi:10.1093/bioinformatics/btv602

6. Flicek P, Amode MR, Barrell D, Beal K, Billis K, Brent S, Carvalho-Silva D, Clapham P, Coates G, Fitzgerald S, Gil L, Giron CG, Gordon L, Hourlier T, Hunt S, Johnson N, Juettemann T, Kahari AK, Keenan S, Kulesha E, Martin FJ, Maurel T, McLaren WM, Murphy DN, Nag R, Overduin B, Pignatelli M, Pritchard B, Pritchard E, Riat HS, Ruffier M, Sheppard D, Taylor K, Thormann A, Trevanion SJ, Vullo A, Wilder SP, Wilson M, Zadissa A, Aken BL, Birney E, Cunningham F, Harrow J, Herrero J, Hubbard TJ, Kinsella R, Muffato M, Parker A, Spudich G, Yates A, Zerbino DR, Searle SM (2014) Ensembl 2014. Nucleic acids research 42:D749-755. doi:10.1093/nar/gkt1196

7. Gudbjartsson DF, Jonasson K, Frigge ML, Kong A (2000) Allegro, a new computer program for multipoint linkage analysis. Nature genetics 25:12-13. doi:10.1038/75514

8. Hoppe T, Cassata G, Barral JM, Springer W, Hutagalung AH, Epstein HF, Baumeister R (2004) Regulation of the myosin-directed chaperone UNC-45 by a novel E3/E4-multiubiquitylation complex in C. elegans. Cell 118:337-349. doi:10.1016/j.cell.2004.07.014

9. Huang N, Lee I, Marcotte EM, Hurles ME (2010) Characterising and predicting haploinsufficiency in the human genome. PLoS genetics 6:e1001154. doi:10.1371/journal.pgen.1001154

10. Ioannidis NM, Rothstein JH, Pejaver V, Middha S, McDonnell SK, Baheti S, Musolf A, Li Q, Holzinger E, Karyadi D, Cannon-Albright LA, Teerlink CC, Stanford JL, Isaacs WB, Xu J, Cooney KA, Lange EM, Schleutker J, Carpten JD, Powell IJ, Cussenot O, Cancel-Tassin G, Giles GG, MacInnis RJ, Maier C, Hsieh CL, Wiklund F, Catalona WJ, Foulkes WD, Mandal D, Eeles RA, Kote-Jarai Z, Bustamante CD, Schaid DJ, Hastie T, Ostrander EA, Bailey-Wilson JE, Radivojac P, Thibodeau SN, Whittemore AS, Sieh W (2016) REVEL: An Ensemble Method for Predicting the Pathogenicity of Rare Missense Variants. American journal of human genetics 99:877-885. doi:10.1016/j.ajhg.2016.08.016

11. Itan Y, Shang L, Boisson B, Patin E, Bolze A, Moncada-Vélez M, Scott E, Ciancanelli MJ, Lafaille FG, Markle JG, Martinez-Barricarte R, de Jong SJ, Kong X-F, Nitschke P, Belkadi A, Bustamante J, Puel A, Boisson-Dupuis S, Stenson PD, Gleeson JG, Cooper DN, Quintana-Murci L, Claverie J-M, Zhang S-Y, Abel L, Casanova J-L (2015) The human gene damage index as a gene-level approach to prioritizing exome variants. Proceedings of the National Academy of Sciences of the United States of America 112:13615-13620. doi:10.1073/pnas.1518646112

12. Kaur G, Lakkaraju A (2018) Early Endosome Morphology in Health and Disease. Advances in experimental medicine and biology 1074:335-343. doi:10.1007/978-3-319-75402-4_41

13. Kumar P, Henikoff S, Ng PC (2009) Predicting the effects of coding non-synonymous variants on protein function using the SIFT algorithm. Nature protocols 4:1073-1081. doi:10.1038/nprot.2009.86

14. Landsverk ML, Li S, Hutagalung AH, Najafov A, Hoppe T, Barral JM, Epstein HF (2007) The UNC-45 chaperone mediates sarcomere assembly through myosin degradation in Caenorhabditis elegans. The Journal of cell biology 177:205-210. doi:10.1083/jcb.200607084

15. Lee CF, Hauenstein AV, Fleming JK, Gasper WC, Engelke V, Sankaran B, Bernstein SI, Huxford T (2011) X-ray crystal structure of the UCS domain-containing UNC-45 myosin chaperone from Drosophila melanogaster. Structure 19:397-408. doi:10.1016/j.str.2011.01.002

16. Liu W, Xie Y, Ma J, Luo X, Nie P, Zuo Z, Lahrmann U, Zhao Q, Zheng Y, Zhao Y, Xue Y, Ren J (2015) IBS: an illustrator for the presentation and visualization of biological sequences. Bioinformatics (Oxford, England) 31:3359-3361. doi:10.1093/bioinformatics/btv362

17. Liu X, Wu C, Li C, Boerwinkle E (2016) dbNSFP v3.0: A One-Stop Database of Functional Predictions and Annotations for Human Nonsynonymous and Splice-Site SNVs. Human mutation 37:235-241. doi:10.1002/humu.22932

18. McKenna A, Hanna M, Banks E, Sivachenko A, Cibulskis K, Kernytsky A, Garimella K, Altshuler D, Gabriel S, Daly M, DePristo MA (2010) The Genome Analysis Toolkit: a MapReduce framework for analyzing next-generation DNA sequencing data. Genome research 20:1297-1303. doi:10.1101/gr.107524.110

19. Petrovski S, Wang Q, Heinzen EL, Allen AS, Goldstein DB (2013) Genic intolerance to functional variation and the interpretation of personal genomes. PLoS genetics 9:e1003709. doi:10.1371/journal.pgen.1003709

20. Preising MN, Gorg B, Friedburg C, Qvartskhava N, Budde BS, Bonus M, Toliat MR, Pfleger C, Altmuller J, Herebian D, Beyer M, Zollner HJ, Wittsack HJ, Schaper J, Klee D, Zechner U, Nurnberg P, Schipper J, Schnitzler A, Gohlke H, Lorenz B, Haussinger D, Bolz HJ (2019) Biallelic mutation of human SLC6A6 encoding the taurine transporter TAUT is linked to early retinal degeneration. FASEB journal : official publication of the Federation of American Societies for Experimental Biology 33:11507-11527. doi:10.1096/fj.201900914RR

21. Ramanathan HN, Ye Y (2012) The p97 ATPase associates with EEA1 to regulate the size of early endosomes. Cell Res 22:346-359. doi:10.1038/cr.2011.80

22. Rao AR, Nelson SF (2018) Calculating the statistical significance of rare variants causal for Mendelian and complex disorders. BMC Med Genomics 11:53-53. doi:10.1186/s12920-018-0371-9

23. Rentzsch P, Witten D, Cooper GM, Shendure J, Kircher M (2019) CADD: predicting the deleteriousness of variants throughout the human genome. Nucleic acids research 47:D886-d894. doi:10.1093/nar/gky1016

24. Schulze A, Lindner M, Kohlmuller D, Olgemoller K, Mayatepek E, Hoffmann GF (2003) Expanded newborn screening for inborn errors of metabolism by electrospray ionization-tandem mass spectrometry: results, outcome, and implications. Pediatrics 111:1399-1406. doi:10.1542/peds.111.6.1399

25. Schweitzer JK, Krivda JP, D'Souza-Schorey C (2009) Neurodegeneration in Niemann-Pick Type C disease and Huntington's disease: impact of defects in membrane trafficking. Current drug targets 10:653-665. doi:10.2174/138945009788680437

26. Sherry ST, Ward MH, Kholodov M, Baker J, Phan L, Smigielski EM, Sirotkin K (2001) dbSNP: the NCBI database of genetic variation. Nucleic acids research 29:308-311

27. Sheth N, Roca X, Hastings ML, Roeder T, Krainer AR, Sachidanandam R (2006) Comprehensive splice-site analysis using comparative genomics. Nucleic acids research 34:3955-3967. doi:10.1093/nar/gkl556

28. Stenson PD, Ball EV, Howells K, Phillips AD, Mort M, Cooper DN (2009) The Human Gene Mutation Database: providing a comprehensive central mutation database for molecular diagnostics and personalized genomics. Human genomics 4:69-72

29. Waterhouse AM, Procter JB, Martin DM, Clamp M, Barton GJ (2009) Jalview Version 2--a multiple sequence alignment editor and analysis workbench. Bioinformatics (Oxford, England) 25:1189-1191. doi:10.1093/bioinformatics/btp033

30. Wohlgemuth SL, Crawford BD, Pilgrim DB (2007) The myosin co-chaperone UNC-45 is required for skeletal and cardiac muscle function in zebrafish. Developmental biology 303:483-492. doi:10.1016/j.ydbio.2006.11.027

31. Wunderlich G, Brunn A, Daimaguler HS, Bozoglu T, Fink GR, Lehmann HC, Weis J, Cirak S (2018) Long term history of a congenital core-rod myopathy with compound heterozygous mutations in the Nebulin gene. Acta myologica : myopathies and cardiomyopathies : official journal of the Mediterranean Society of Myology 37:121-127

32. Yeo G, Burge CB (2004) Maximum entropy modeling of short sequence motifs with applications to RNA splicing signals. Journal of computational biology : a journal of computational molecular cell biology 11:377-394. doi:10.1089/1066527041410418
